# A Subject-Independent Temporal Framework for Behavioural Eye-Based Driver Drowsiness Detection

**DOI:** 10.3390/s26196193

**Published:** 2026-09-30

**Authors:** Olusola Olajide Ajayi, Anish Mathew Kurien, Karim Djouani, Lamine Dieng

**Affiliations:** 1 F’SATI, French South African Institute of Technology, Tshwane University of Technology, Pretoria 0183, South Africa; kurienam@tut.ac.za (A.M.K.); djouani@gmail.com (K.D.); lamine.dieng@univ-eiffel.fr (L.D.); 2 LISSI Laboratory, Université Paris-Est Créteil, France, and, F’SATI, French South African Institute of Technology, Tshwane University of Technology, Pretoria 0183, South Africa; 3 MAST Laboratory, Université Gustave Eiffel, All. Des Ponts et Chaussees, 44340 Bouguenais, France and, F’SATI, French South African Institute of Technology, Tshwane University of Technology, Pretoria 0183, South Africa

**Keywords:** driver drowsiness detection, eye aspect ratio, behavioural features, subject independent evaluation, leave one driver out, random forest, LSTM, intelligent transportation systems

## Abstract

Driver drowsiness detection systems are often evaluated using data partitions that may retain observations from the same individuals across training and testing, making their ability to generalise to unseen drivers difficult to assess. This study investigates behavioural eye-based drowsiness detection under strict subject-independent evaluation using 65,929 facial image frames from four drivers in the NTHU Driver Drowsiness Detection dataset. Eye Aspect Ratio (EAR), Rolling Mean EAR, EAR Delta and an Eye Closure Indicator were represented over temporal windows of 10, 20 and 30 frames and evaluated using Logistic Regression, Random Forest, Long Short-Term Memory and Bidirectional Long Short-Term Memory classifiers under Leave-One-Driver-Out validation. Logistic Regression achieved the highest mean accuracy, whereas Random Forest at 30 frames produced the highest drowsy-class F1-score and Area under the Precision–Recall Curve. Controlled baseline analysis showed that temporal context contributed more substantially to performance than the additional engineered EAR features, while recurrent architectures did not consistently outperform the classical classifiers. Performance also varied across unseen drivers, and the modest absolute accuracy cautions against interpreting the results as evidence of deployment-ready detection. The study demonstrates the importance of separating driver identities during evaluation and provides a reproducible framework for examining temporal behavioural representations under unseen-driver conditions.

## 1. Introduction

### 1.1. Motivation and Safety Context

Driver drowsiness is a major road-safety risk that contributes to traffic crashes, injuries, and fatalities worldwide. In 2023 alone, driver fatigue was a contributing factor in almost 91,000 collisions in the United States, which led to over 800 fatalities, according to the National Highway Traffic Safety Administration (NHTSA) [1]. The severity of fatigue is similar to alcohol-impaired driving since it impairs important driving functions such as attention, reaction time, and situational awareness [2].

Driver monitoring systems aim to reduce this risk by detecting behavioural or physiological indicators of fatigue before they contribute to unsafe driving. Generally speaking, detection techniques fall into three categories: behavioural (such as eye blink rate and facial expressions), physiological (such as EEG and ECG), and vehicular (such as steering wheel motion and lane departure) [3,4]. Among these, behavioural-based methods have become very popular since they are non-invasive, work well for real-time applications, and work with little hardware [5].

### 1.2. Behavioural Eye-Based Detection and Temporal Modeling

In the behavioural realm, eye-related indicators are widely used because they provide non-invasive and computationally accessible representations of driver alertness. The Eye Aspect Ratio (EAR), PERCLOS (% of eye closure), and blink length are among the metrics commonly used to assess drowsiness [6,7] EAR was introduced by [8] as a computationally efficient measure of eye openness based on the geometric relationship among six periocular landmarks. Its continued relevance to real-time driver drowsiness detection has been demonstrated in recent studies employing EAR to monitor eyelid closure and variations in driver alertness [9].

The temporal progression of drowsiness is frequently missed by static, frame-by-frame methods, which do not take into consideration how drowsiness develops across sequences of frames [10,11]. Since fatigue is a progressive, dynamic condition that changes over time, sequence-learning models are necessary. Deep learning techniques, particularly recurrent architectures such as Long Short-Term Memory (LSTM) networks, have increasingly been applied to model temporal dependencies in driver drowsiness detection [12,13]. It has been demonstrated that these models increase prediction accuracy in sequence-based problems and are made to handle time-dependent patterns [14]. Despite these advantages, the effectiveness of temporal architectures remains dependent on the adopted behavioural representation and evaluation protocol.

### 1.3. Limitations of Existing Evaluation Protocols

Despite the high classification accuracies reported in recent driver drowsiness detection studies ([15,16,17]), the reliability of reported performance depends considerably on the adopted evaluation protocol. In particular, frame level or participant dependent data partitioning may allow observations from the same driver to appear in both training and testing sets, potentially enabling models to learn subject specific characteristics rather than generalisable fatigue patterns.

This concern is particularly relevant to behavioural drowsiness detection because eye geometry, blinking behaviour, facial structure, and eyelid dynamics naturally vary among individuals. Consequently, performance obtained when the training and testing samples share driver identities may not adequately represent deployment conditions in which a monitoring system encounters previously unseen drivers.

Furthermore, fatigue develops progressively over time, whereas several existing image-based approaches primarily classify individual frames without explicitly preserving temporal behavioural information. These limitations highlight two related research needs: temporal representation of behavioural fatigue progression and rigorous evaluation under complete subject separation. Accordingly, this study investigates derived eye-based temporal features, whereby Rolling Mean EAR, EAR Delta, and the Eye Closure Indicator are derived from the raw EAR measurements to characterise temporal changes in eye behaviour, under a strict subject independent evaluation protocol designed to assess generalisation to unseen drivers.

### 1.4. Contributions of This Study

This study makes the following contributions to behavioural driver drowsiness detection research:

i. A strict subject-independent evaluation design based on Leave-One-Driver-Out validation, ensuring complete separation between the training and test drivers and reducing the risk of identity leakage.
ii. A compact behavioural feature representation combining the raw Eye Aspect Ratio, rolling mean EAR, frame-to-frame EAR variation, and a binary eye-closure indicator.
iii. A controlled comparison of the Logistic Regression, Random Forest, LSTM, and BiLSTM models using identical feature sequences, sequence lengths, and unseen-driver evaluation conditions.
iv. An empirical investigation of three temporal sequence lengths of 10, 20, and 30 frames to determine how temporal context affects classical and recurrent classifiers.

### 1.5. Paper Organization

The remainder of this paper is organised as follows. Section 2 reviews behavioural, image-based, temporal, and subject-independent approaches to driver drowsiness detection. Section 3 describes the dataset, feature engineering, temporal sequence construction, classification models, experimental configuration, and Leave-One-Driver-Out evaluation protocol. Section 4 presents and discusses the experimental results including model comparison, sequence-length effects, subject-wise performance, and comparison with previous studies. Section 5 concludes the paper, outlines its limitations, and identifies directions for future research.

## 2. Related Work

### 2.1. Image-Based Deep Learning Approaches

Earlier image-based studies provide important context for the progression of driver drowsiness detection from computationally constrained CNN and facial feature approaches towards more recent lightweight and mobile oriented architectures. In [18], a compressed deep neural network utilizing facial landmarks was deployed on a Jetson TK1, achieving 14.9 FPS and 89.5% accuracy. The original 4-stream model was distilled into a smaller, faster 2-stream “Compressed-2 Model” (10 MB), highlighting the trade-off between speed and accuracy for embedded systems. Meanwhile, [19] developed an Intelligent Driver Warning System using CNNs and a Local Octal Pattern feature map to detect driver mental states through facial expressions, addressing accuracy issues in faulty data, though specific limitations of the system were not detailed.

The research in [20] used facial and ocular features with Viola-Jones, PCA, and an SVM classifier to achieve 98% accuracy in fatigue detection. In [21], an ensemble of four CNNs (AlexNet, VGG-FaceNet, FlowImageNet, ResNet) reached 85% accuracy, highlighting neural network sensitivity to training data. The authors in [22] proposed a fatigue detection method combining a cumulative time-effect model with a BP neural network using EAR, MAR, and head position, improving robustness but was challenged by false positives from natural behaviors. In [23], “Sleepy Wheels” used an EfficientNetV2B0-BlazeFace ensemble to detect drowsiness in real-time with 97% accuracy on mobile devices, even under partial occlusion. The study in [24] developed a CNN-based system in MATLAB to monitor facial cues and prevent fatigue-related accidents. In [25], an image processing system using PERCLOS, AdaBoost, and Hough transform accurately detected eye states under varied conditions, but struggled with sunglasses, side angles, and lacked embedded integration.

In [26], the authors came up with a real-time CNN and MobileNet SSD-based system to detect driver distraction, drowsiness, and seat belt use with 97% accuracy, while Xing et al. [27] demonstrated the application of deep learning to driver activity recognition for intelligent vehicles. In [28], the authors used CNNs for facial landmark detection and eye state analysis, achieving 94% accuracy, though performance dropped in poor lighting. The work in [29] showed that data augmentation significantly improved the DL model accuracy for drowsiness detection, especially under challenging conditions. The authors in [30] introduced a MAX–MIN adaptive method using ShuffleNet V2K16 and Dlib for robust facial feature analysis, achieving high precision (98.8%) and F1-score (94.3%). In [15], the authors demonstrated that EfficientNetB7 outperformed other models with 99.87% accuracy in eye-image-based fatigue detection, confirming DL’s effectiveness for road safety.

In [31], a lightweight and efficient deep learning model based on MobileNetV2 was proposed using the UTA-RLDD dataset. With an accuracy of 94.03%, a compact size of 8.95 MB, and a training time of just 60.6 s, the model proved highly suitable for real-time mobile deployment. The work in [16] offered a real-time drowsiness detection system combining Convolutional Neural Networks (CNNs), Haar Cascade, InceptionV3, and MobileNetV2 through transfer learning. Using multiple merged datasets, the system achieved classification accuracies ranging from 90% to 99.86% in both binary and multi-class scenarios, emphasizing its practical application in mobile environments. In [32], a CNN-based model outperformed RNNs and custom neural networks on two Kaggle datasets by detecting eye states (open or closed), showing the effectiveness of CNNs for real-time fatigue monitoring. The authors in [33] analyzed facial features, specifically Eye Aspect Ratio (EAR), Mouth Aspect Ratio (MAR), and head posture, using VGG16 and Random Forest algorithms. It attained 97.2% accuracy but noted challenges due to lighting variations and reliance on a simulated driving environment. In [34], a CNN-based model used EAR, MAR, and Percentage of Eye Closure (PERCLOS) to detect drowsiness with a 98.7% accuracy rate. However, it faced limitations such as sensitivity to lighting changes and head pose shifts. The work in [35] presented a CNN trained on a labeled dataset to classify drowsiness, achieving a true positive rate of 95%, a true negative rate of 98%, and an overall accuracy of 92.4%. The study highlighted challenges in computational efficiency and generalization for real-time, in-vehicle use. Finally, [17] developed a real-time monitoring system using deep learning models, InceptionV3, Xception, and DenseNet121 with transfer learning, to identify indicators like head tilting, eye closure, and yawning from video and image data. Based on an augmented Kaggle dataset with 2904 images, the Xception model performed best with a 99.36% accuracy. The system provided immediate driver alerts, underscoring its real-world applicability for road safety.

### 2.2. Behavioural and Landmark-Based Methods

In [36], the authors developed a real-time fuzzy expert system using video analysis, Kalman filters, and skin detection to monitor yawning and eye closure for driver fatigue detection, achieving high accuracy despite unspecified limitations. In [37], the “DriveSafe” iPhone app combined computer vision and phone sensors to detect driver distraction and fatigue, achieving 82% accuracy and 92% recall, though it faced challenges like low camera resolution and data collection issues. In [38], the authors introduced a real-time Android-based solution using EAR and ECR metrics, facial landmarks, and a Random Forest classifier, reaching 84% accuracy without expensive sensors. Drowsiness triggers alerts when EAR < 0.25 or ECR > 0.5, with potential expansion into ride-sharing applications.

In [39], a hybrid GWO–ANN model classified facial and eye features (e.g., PERCLOS, blink frequency), outperforming other optimization methods in real-time detection. The authors in [40] suggested a vision-based drowsiness detection method using Support Vector Machines (SVMs) and spatiotemporal facial features extracted with LBP-TOP. It achieved 82% and 90% accuracy on the UTA-RLDD and DROZY datasets, respectively, offering a non-invasive and robust alternative to physiological systems. In [41], a fuzzy logic-based system combined enhanced Viola-Jones facial feature detection with multi-fatigue indicators, achieving 97.5% accuracy in real-time tests. Despite strong performance, challenges such as lighting variability and feature occlusion were noted.

### 2.3. Temporal Modeling Approaches

Driver drowsiness is a progressive behavioural state that evolves over time rather than instantaneously. Consequently, temporal modelling techniques have attracted increasing attention because they are capable of learning sequential behavioural patterns associated with fatigue progression. Unlike conventional frame-based classifiers that analyse individual images independently, temporal models exploit the relationships between consecutive observations to capture sustained eye closure, blink dynamics, and gradual behavioural transitions.

The work in [42] proposed a CNN–LSTM architecture for driver drowsiness prediction by combining spatial feature extraction with temporal sequence learning. Using eye state and yawning information, the model achieved a reported classification accuracy of 97.27%, demonstrating the potential of integrating convolutional and recurrent networks for fatigue recognition. In [43], the authors framed drowsiness as a temporal action detection problem, using a two-stream multi-feature network with CLAHE and optical flow to enhance robustness under challenging lighting conditions. Similarly, the study in [13] employed Long Short-Term Memory (LSTM) networks to analyse temporal sequences of eye and mouth images collected over time. The proposed recurrent framework attained an accuracy of 97.25%, further illustrating the capability of LSTM architectures to model behavioural fatigue progression.

More recently, Ref. [44] applied a Video Vision Transformer (ViViT) to the NTHU-DDD dataset to jointly capture spatial and temporal information from video sequences, reporting 96.2% accuracy and 95.9% F1-score. Their approach analysed eye conditions and head movements directly from video and employed a 70:15:15 training, validation, and test split, with threefold cross-validation applied to the training data. While these results demonstrate the potential of transformer-based temporal modelling for driver drowsiness detection, the reported evaluation did not employ an explicitly subject-independent partitioning protocol. Consequently, its performance is not directly comparable with the results obtained under leave-one-driver-out evaluation, where the test driver is completely unseen during model development.

Despite these promising results, most existing temporal studies have primarily focused on improving predictive accuracy, while comparatively less attention has been given to the influence of evaluation methodology on reported performance. However, a realistic assessment of temporal models also requires validation protocols that preserve subject independence and temporal consistency. Consequently, both temporal modelling and rigorous evaluation design should be considered complementary requirements for developing robust behavioural driver drowsiness detection systems.

### 2.4. Evaluation Protocol Realism and Identity Leakage Risks

Although many recent deep learning models report classification accuracies exceeding 90% for driver drowsiness detection (e.g., [15,16,17]), the reported performance is strongly influenced by the adopted evaluation protocol. In many published studies, datasets are partitioned at the frame level rather than at the subject level, allowing observations from the same individual to appear in both the training and testing sets. While this strategy increases the number of available training samples and often improves the reported accuracy, it may inadvertently introduce subject identity leakage, whereby classifiers partially learn driver-specific facial characteristics instead of fatigue-related behavioural patterns.

This concern is particularly important for behavioural fatigue detection because facial geometry, blink frequency, eyelid movement, and eye-opening characteristics vary naturally across individuals. Consequently, models evaluated using participant-dependent or random frame-level partitions may produce overly optimistic estimates of performance that do not accurately reflect deployment conditions involving previously unseen drivers. Similar concerns regarding temporal coherence and realistic evaluation have been highlighted in benchmark temporal modelling studies such as [11].

Recent studies further demonstrate the importance of rigorous subject-independent evaluation. Ref. [45] reported that participant-independent validation provides a more reliable assessment of drowsiness detection systems because all observations belonging to the test participant remain completely unseen during model development. Likewise, Ref. [46] demonstrated that classification performance decreases substantially when evaluation progresses from intra-session to cross-session and ultimately to cross-subject validation, illustrating the increased difficulty of recognising fatigue in previously unseen individuals. These findings suggest that the evaluation strategy is as important as model architecture when assessing the practical applicability of behavioural fatigue detection systems.

In real-world intelligent transportation environments, driver monitoring systems must operate reliably across diverse users, driving conditions, and recording sessions. Accordingly, realistic evaluation protocols should preserve temporal coherence, eliminate subject overlap between training and testing partitions, and assess performance using completely unseen drivers. Such protocols provide a more conservative but substantially more reliable estimate of model generalisation capability.

The literature reviewed demonstrates significant progress in behavioural driver drowsiness detection through advances in image-based learning, behavioural feature extraction, and temporal modelling techniques. Nevertheless, three important gaps remain. First, many reported performance results are obtained using participant-dependent or frame-level evaluation protocols that may overestimate model generalisation. Second, relatively few studies have investigated behavioural temporal representations under strict subject-independent validation. Third, limited work has systematically examined how both conventional machine learning and recurrent deep learning models perform under identical behavioural feature representations and unseen-driver evaluation conditions. These limitations motivate the subject-independent temporal framework proposed in this study, which combines engineered behavioural eye features with rigorous Leave-One-Driver-Out evaluation to provide a realistic assessment of driver drowsiness detection performance.

## 3. Methodology

### 3.1. Overall Research Framework

This study proposes a subject-independent temporal framework for behavioural driver drowsiness detection using EAR-based behavioural features extracted from facial images. The framework was designed to evaluate whether temporal modelling of behavioural eye dynamics can improve generalisation to previously unseen drivers under realistic deployment conditions. Unlike conventional evaluation strategies that randomly partition image frames irrespective of driver identity, the proposed framework adopts a Leave-One-Driver-Out (LODO) validation protocol to eliminate subject identity leakage and provide a more reliable assessment of model performance.

**Figure 1:**
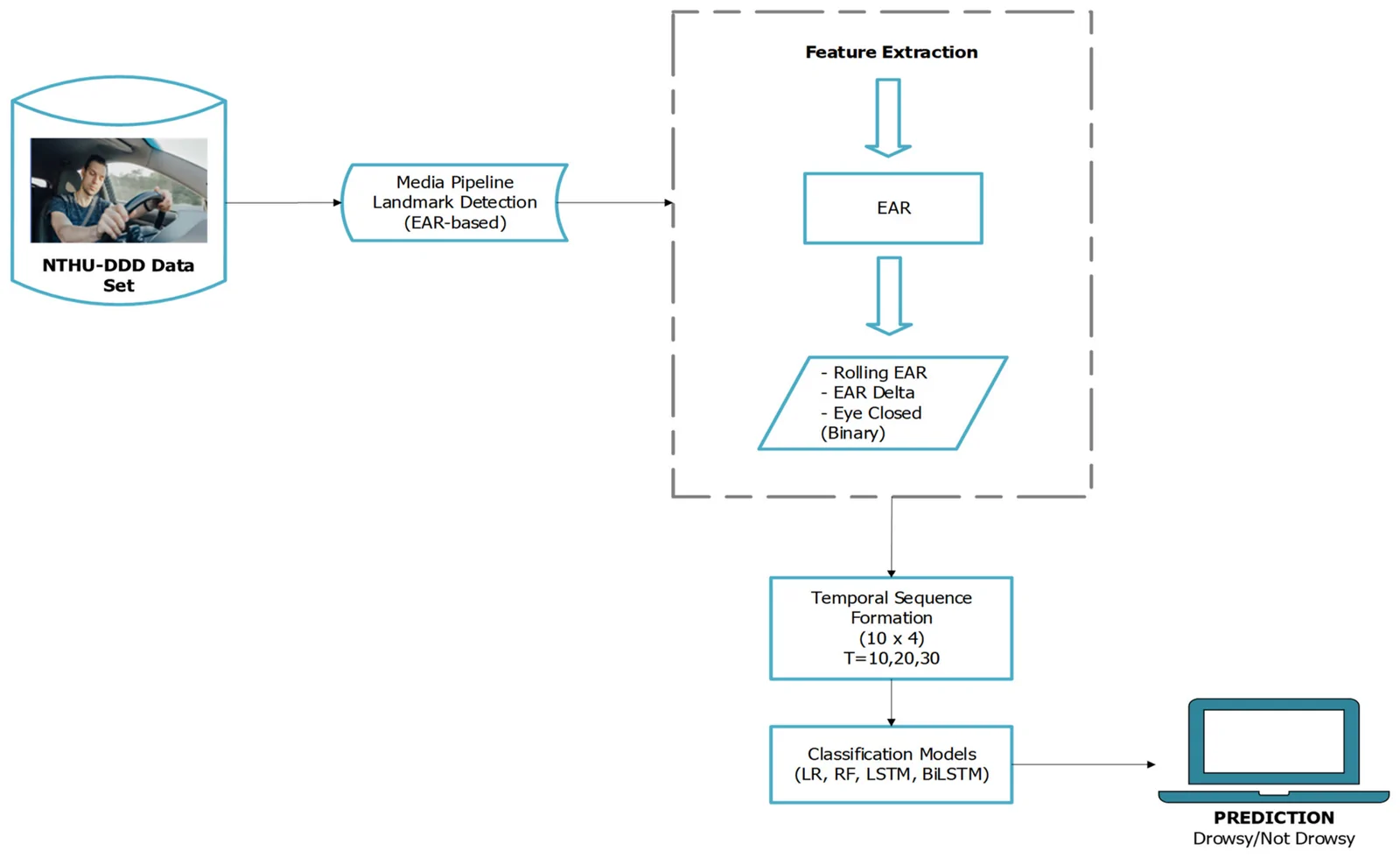
Methodological workflow.

The methodological pipeline comprises six sequential stages, as illustrated in Figure 1. First, behavioural eye features were extracted from the NTHU Driver Drowsiness Detection (NTHU-DDD) dataset using MediaPipe facial landmark detection. Second, the Eye Aspect Ratio (EAR) was computed for each frame and further enriched through temporal feature engineering by deriving the rolling mean EAR, frame-to-frame EAR variation, and a binary eye-closure indicator. Third, the engineered frame-level features were organised into fixed-length temporal sequences of varying lengths to capture the progressive nature of driver fatigue.

In the fourth stage, the generated sequences were partitioned using a subject-independent Leave-One-Driver-Out protocol in which all samples belonging to one driver were reserved exclusively for testing while the remaining drivers were used for training. This evaluation strategy was repeated until every driver had served once as the unseen test subject, thereby simulating real-world deployment where driver monitoring systems encounter individuals not observed during model development.

The fifth stage involved the implementation of four classification models representing both classical machine learning and temporal deep learning approaches. Logistic Regression and Random Forest were selected as classical baseline classifiers, while Long Short-Term Memory (LSTM) and Bidirectional Long Short-Term Memory (BiLSTM) networks were employed to model temporal dependencies within the behavioural eye sequences. All models were trained and evaluated under identical experimental conditions to ensure fair comparison.

Finally, model performance was assessed using multiple complementary evaluation metrics, including Accuracy, Precision, Recall, F1-score, Area under the Receiver Operating Characteristic Curve (AUROC), and Area under the Precision–Recall Curve (AUPRC). Performance statistics were computed independently for each unseen driver and subsequently aggregated to provide an overall assessment of model robustness and generalisation capability. The complete methodological workflow adopted in this study is illustrated in Figure 1.

### 3.2. Dataset Description and Available Subjects

The behavioural eye data used in this study were obtained from the NTHU Driver Drowsiness Detection (NTHU-DDD) dataset [47], a widely used benchmark dataset for driver drowsiness research. The dataset contains facial image sequences captured under realistic driving scenarios involving variations in illumination, facial accessories, and driver conditions. These variations make the dataset suitable for evaluating the robustness of behavioural fatigue detection algorithms under practical driving environments.

The binary target labels used in this study were inherited from the drowsy and non-drowsy categories of the downloaded NTHU-DDD source subset. During data preparation, images obtained from the drowsy category were assigned the target label 1, whereas those from the non-drowsy category were assigned the target label 0. These target labels were not generated from the EAR measurements or from the EAR-based Eye Closure Indicator subsequently derived during feature extraction. Thus, the target class assignment and the EAR-based predictor construction were separate stages of the experimental pipeline.

The downloaded NTHU-DDD source subset available for this study contained recordings from four drivers: Subjects 001, 002, 005, and 006. Further inspection of the original source files confirmed that Subjects 003 and 004 were not present in either the drowsy or non-drowsy image collections used in this study. Accordingly, these subjects were not excluded during preprocessing, temporal sequence construction, or model evaluation; rather, all four subjects represented in the available source subset were retained for analysis. The resulting dataset comprised 65,929 facial image frames from 27 driving video streams. During preprocessing, each image filename was automatically parsed to extract the corresponding subject identifier, driving scenario, and frame number, thereby preserving the chronological order of every driving sequence throughout the experimental pipeline.

The experimental dataset exhibited a moderate class imbalance, consisting of 36,023 drowsy frames (54.64%) and 29,906 non-drowsy frames (45.36%). Rather than applying artificial balancing techniques such as oversampling or synthetic data generation, the original class distribution was preserved to ensure that the trained models reflected naturally occurring behavioural patterns and to avoid introducing synthetic bias into the evaluation process. Table 1 summarises the characteristics of the dataset used in this study.

To further illustrate the distribution of the experimental data, Table 2 presents the number of drowsy and non-drowsy samples contributed by each subject. The noticeable variation in class distribution across drivers reflects the inherent diversity of behavioural fatigue manifestations among individuals and provides a basis for evaluating model generalisation under subject-independent conditions.

The adoption of a subject-independent evaluation protocol was a deliberate methodological choice. Unlike conventional random train–test partitioning, which may inadvertently include samples from the same driver in both training and testing sets, the Leave-One-Driver-Out strategy ensures the complete separation of driver identities. Consequently, the reported performance more accurately reflects the capability of the proposed framework to generalise to previously unseen drivers, thereby providing a more rigorous assessment of its practical applicability in intelligent transportation systems.

### 3.3. Behavioural Feature Extraction

Behavioural fatigue indicators were extracted from each facial image using the MediaPipe Face Mesh framework, which provides dense facial landmark localisation in real-time. MediaPipe was selected due to its computational efficiency, robustness to moderate head movements, and ability to accurately detect eye landmarks under varying illumination and facial appearance conditions. For each image frame, the detected facial landmarks corresponding to both eyes were used to compute the Eye Aspect Ratio (EAR), a geometric descriptor widely employed for monitoring eye closure and blink behaviour during driver fatigue assessment.

The EAR was computed from six eye landmarks according to Equation (1), where the vertical eye distances are normalised by the horizontal eye width.

(1) $$\mathrm{EAR}=\frac{\left|\left|p2-p6\right|\right|+\left|\left|p3-p5\right|\right|}{2*\left|\left|p1-p4\right|\right|}\;$$

where:

*p*_1_……*p*_6_ denote the six facial landmarks defining the eye contour, while

∥⋅∥ represents the Euclidean distance between two landmark points.

Higher EAR values generally indicate an open eye state, whereas sustained reductions in EAR correspond to progressive eye closure associated with drowsiness. To improve the robustness of behavioural fatigue representation beyond the raw Eye Aspect Ratio, three additional temporal features were derived from the EAR sequence. These engineered features capture gradual behavioural trends, rapid frame-to-frame variations, and sustained eye closure associated with driver drowsiness.

The Rolling Mean EAR was computed over a sliding window of five consecutive frames to smooth transient fluctuations while preserving longer-term behavioural changes:

(2) $${\mathrm{EAR}}_{\mathrm{RM}(t)}\;=1/5\sum_{i=t-4}^{t}\mathrm{EARi}\;$$

where:

EAR_RM(t)_ denotes the rolling mean Eye Aspect Ratio at frame t.

The frame-to-frame variation in EAR was calculated as the first-order temporal difference:

ΔEAR_t_
*= EAR_t_ − EAR_t−_*_1_ (3)

where:

ΔEAR_t_ denotes the instantaneous change in eye openness between two consecutive frames (*t*, *t* − 1).

A binary Eye Closure Indicator was subsequently generated by assigning a value of 1 when EAR < 0.21 and 0 otherwise. The threshold was fixed a priori in the experimental pipeline and was used solely to construct the eye-closure feature; it was not used to determine the drowsy/non-drowsy target labels. Because EAR thresholds may vary across subjects and acquisition conditions, the robustness of the selected value was subsequently examined through a sensitivity analysis using thresholds from 0.19 to 0.23.

(4) $${\mathrm{EC}}_{t}=\left\{\begin{array}{c} 1,\;\mathrm{if}\;\mathrm{EARt}<0.21\\ \;\;0,\;\mathrm{otherwise} \end{array}\right.$$

where:

EC_t_ = 1 denotes a closed-eye state and EC_t_ = 0 denotes an open-eye state at frame t.

Consequently, each image frame is represented by the following four-dimensional behavioural feature vector:

x_t_ = [EAR_t_, EAR_RM(t)_, ΔEAR_t_, EC_t_] (5)

To capture both instantaneous eye behaviour and its short-term temporal dynamics, four behavioural features were extracted from each image frame. These derived features, together with their descriptions and their respective roles in the proposed framework, are summarised in Table 3.

**Figure 2:**
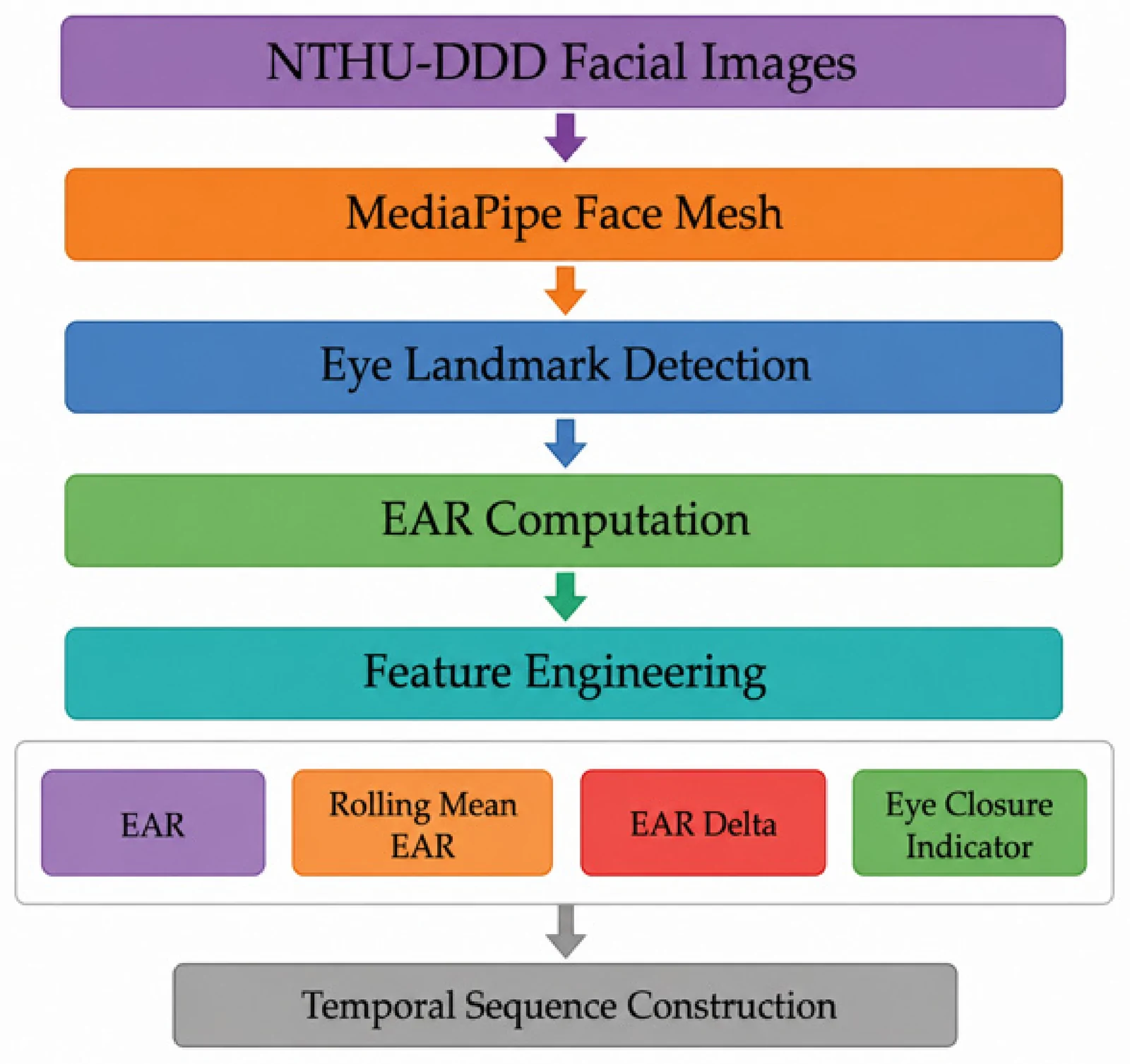
Feature engineering process.

As shown in Table 3, each derived feature contributes complementary information for behavioural fatigue analysis. While the Eye Aspect Ratio (EAR) captures the instantaneous geometric state of the eye, the rolling mean EAR reduces short-term fluctuations and emphasises gradual behavioural changes. Similarly, the EAR Delta quantifies rapid eye movement variations associated with blinking, whereas the Eye Closure Indicator provides a simplified representation of sustained eyelid closure. Collectively, these four features provide a richer temporal description of driver eye behaviour than the EAR alone and constitute the feature representation used for temporal sequence construction and subsequent model development. The complete feature engineering process adopted in this study is illustrated in Figure 2.

### 3.4. Temporal Sequence Construction

Driver fatigue is a progressive behavioural state that evolves over time rather than as an isolated event in a single image frame. Consequently, analysing individual frames independently may fail to capture the temporal patterns associated with the gradual eye closure, blink frequency, and sustained eyelid movements that typically precede drowsiness. To preserve these temporal dependencies, the derived frame-level behavioural features were organised into fixed-length sequential samples prior to model training. Each temporal sequence was constructed by grouping consecutive feature vectors according to:

S_i_ = [x_t–T+1_, x_t−T+2_,…,x_t_] ∈ R^T×4^ (6)

where:

*S_i_* denotes the *i*th temporal sequence;

*x_t_ denotes the four dimensional behavioural feature vector at frame t*;

*T* represents the sequence length, with *T* ∈ {10, 20, 30}.

In this study, three sequence lengths (T = 10, T = 20, and T = 30) were investigated to evaluate the influence of temporal context on subject-independent behavioural fatigue detection.

The temporal sequences were generated using an overlapping sliding-window approach with a stride of one available frame while maintaining the original chronological frame order within each driving stream. The analysed daytime glasses and no-glasses recordings had a nominal frame rate of 30 fps; consequently, sequence lengths of 10, 20 and 30 observations correspond to nominal temporal windows of approximately 0.33, 0.67 and 1.00 s, respectively. An audit of the processed frame indices showed that 65,837 out of 65,902 adjacent observation pairs (99.901%) were strictly consecutive, while 65 pairs (0.099%) contained one or more missing frame indices. Consequently, 99.534%, 99.161%, and 98.804% of the generated T = 10, T = 2,0 and T = 30 windows, respectively, were strictly frame-contiguous. Where an intermediate frame was unavailable in the processed feature series, the sequence construction retained chronological order by proceeding to the next available observation rather than imputing the missing EAR value.

The behavioural label assigned to each temporal sequence corresponded to the label of its final frame. This strategy preserves causal consistency because predictions are based exclusively on present and preceding observations without incorporating information from future frames, thereby preventing temporal information leakage.

**Figure 3:**
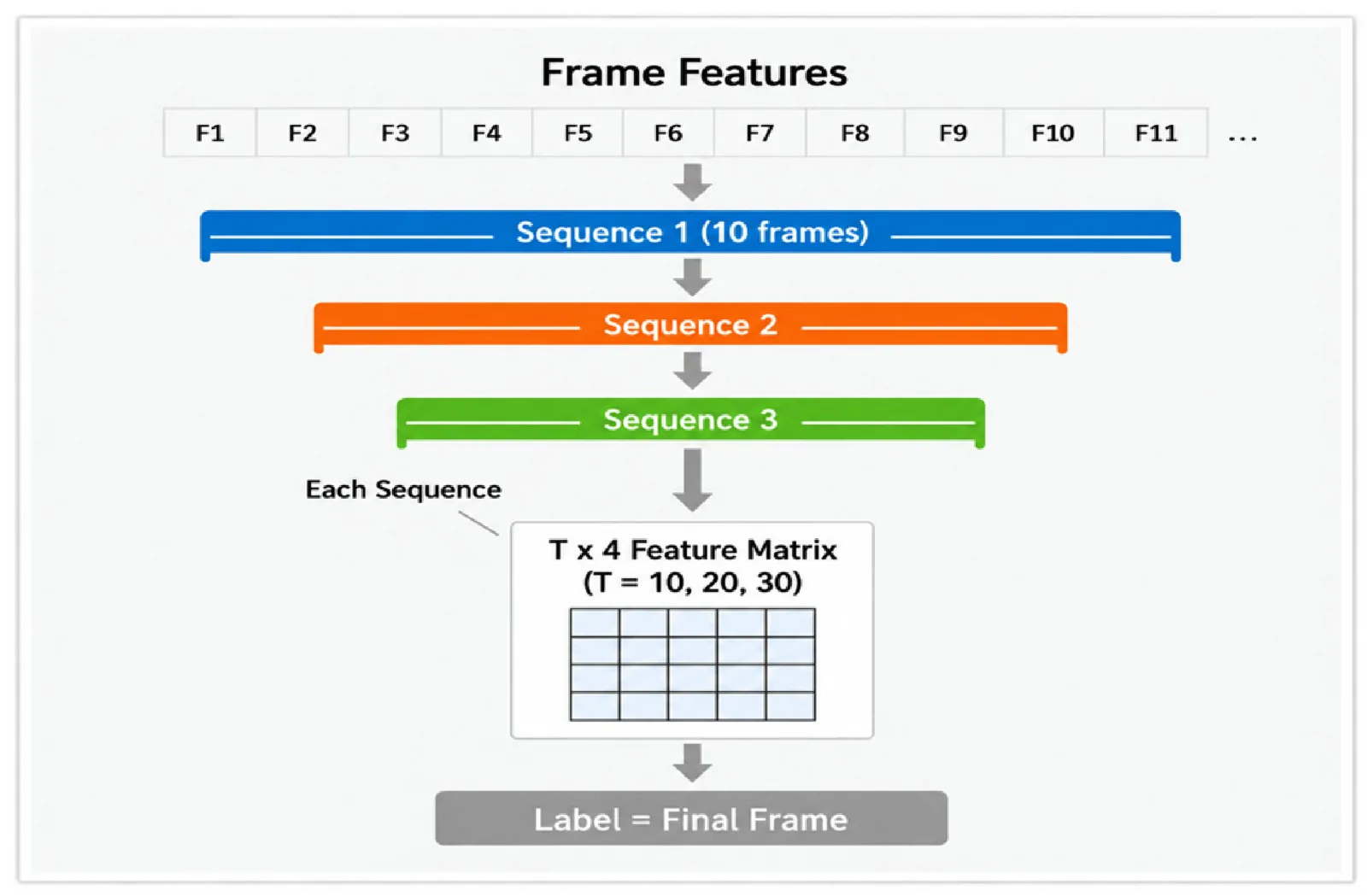
Temporal sequence process.

Sequence generation was performed independently within each driving video stream to ensure that no sequence crossed stream boundaries or mixed observations from different recording sessions. Furthermore, all generated sequences retained the subject identifier associated with their original driving session, thereby facilitating the implementation of the Leave-One-Driver-Out validation protocol described in Section 3.5. Figure 3 illustrates the temporal sequence construction process adopted in this study.

As illustrated in Figure 3, the sliding-window strategy allows consecutive sequences to share temporal information while preserving the natural progression of driver behaviour. This approach enables the learning algorithms to model transitions from alertness to drowsiness rather than relying solely on isolated frame characteristics. By evaluating multiple sequence lengths under identical experimental conditions, the study further investigates the extent to which temporal context contributes to subject-independent behavioural fatigue detection.

### 3.5. Subject-Independent Leave-One-Driver-Out Evaluation

A major challenge in behavioural driver drowsiness detection is ensuring that the reported model performance reflects genuine generalisation rather than the memorisation of driver-specific behavioural patterns. Many previous studies have employed random train–test partitioning in which image frames or temporal sequences originating from the same driver may appear in both the training and testing sets. Although such approaches often produce high classification accuracies, they may inadvertently introduce subject identity leakage, resulting in overly optimistic estimates of model performance.

**Figure 4:**
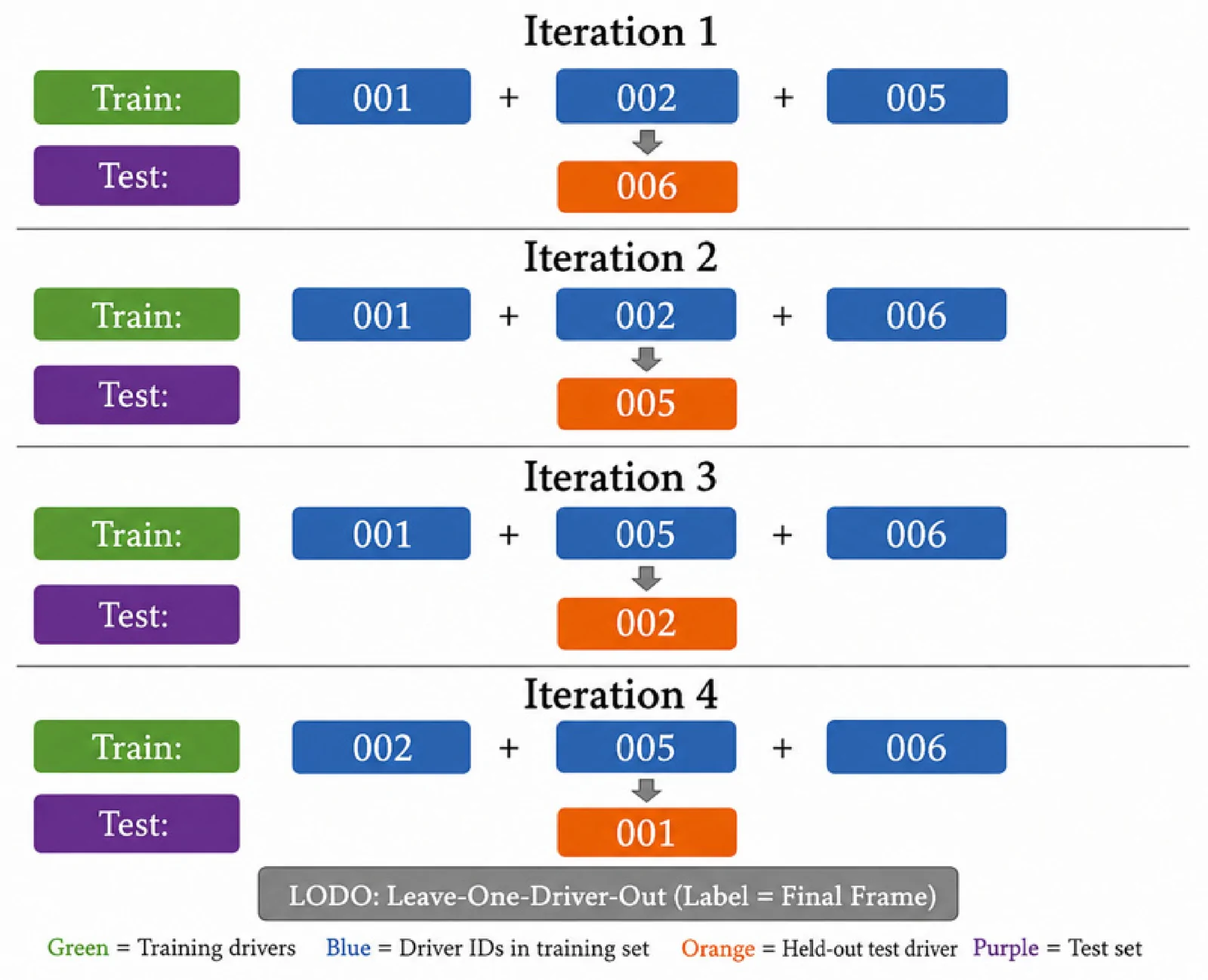
LODO validation technique.

To minimise this source of bias, this study adopted a subject-independent Leave-One-Driver-Out (LODO) validation strategy. Under this protocol, all temporal sequences belonging to one driver were reserved exclusively for testing, while sequences from the remaining three drivers were used for model training. This procedure was repeated four times so that each of the four available drivers (001, 002, 005, and 006) served once as the independent test subject. Consequently, every reported performance metric represents the ability of the trained models to recognise fatigue patterns in drivers that were completely unseen during model development. Figure 4 illustrates the Leave-One-Driver-Out evaluation protocol implemented in this study.

The LODO protocol was adopted to prevent information from the same driver from appearing in both the training and test partitions. Such separation is particularly important in sequential drowsiness data because temporally adjacent observations, overlapping windows, and repeated behavioural patterns from the same individual may be highly correlated. Random sequence-level partitioning may therefore allow the model to exploit driver-specific or recording-specific characteristics rather than learning patterns that generalise across individuals. Previous comparisons of validation strategies have similarly identified Leave-One-Participant-Out evaluation as an appropriate approach for addressing inter-individual variability and autocorrelation in drowsiness data [44]. Accordingly, each fold in the present study represents a genuine unseen-driver test rather than a random holdout of observations from already represented drivers.

Unlike conventional random partitioning, the adopted protocol ensures complete separation between the training and testing subjects throughout the experimental process. Furthermore, temporal sequences were generated independently within each driving stream before subject partitioning, thereby preventing sequence overlap across drivers or driving sessions. This design preserves temporal integrity while approximating the unseen-driver condition encountered by intelligent driver monitoring systems that encounter previously unseen individuals during deployment.

For each fold, model predictions were compared with the corresponding ground truth labels to compute the evaluation metrics described in Section 3.8. The final performance of each classifier was obtained by averaging the results across all four independent test folds. Reporting the mean performance together with its variability across the independent test folds provides a comprehensive assessment of model robustness under cross driver evaluation.

The adopted validation protocol constitutes a more stringent assessment than conventional random train–test evaluation because it eliminates subject identity leakage and directly evaluates the capacity of the proposed framework to generalise across different drivers. Such subject-independent evaluation is particularly important for behavioural fatigue detection systems intended for practical intelligent transportation applications, where the monitored drivers are unlikely to have been observed during model training.

### 3.6. Classification Models

To comprehensively evaluate the effectiveness of the proposed behavioural feature representation under subject-independent conditions, four classification models were investigated. Two conventional machine learning algorithms, namely Logistic Regression (LR) and Random Forest (RF), were implemented as baseline classifiers while two recurrent neural network architectures, Long Short-Term Memory (LSTM) and Bidirectional Long Short-Term Memory (BiLSTM) were employed to model temporal dependencies within the behavioural eye sequences. All models were trained and evaluated using identical temporal sequences and the same Leave-One-Driver-Out validation protocol to ensure a fair and consistent comparison. For the classical machine learning models, each *T* × 4 temporal sequence was reshaped into a one dimensional feature vector of length 4*T*, whereas the LSTM and BiLSTM models retained the original sequential *T* × 4 representation. This allowed all four classifiers to use identical behavioural information while accommodating their respective input requirements.

#### 3.6.1. Logistic Regression

Logistic Regression was employed as a linear baseline classifier owing to its simplicity, computational efficiency, and widespread use in binary classification problems. The model estimates the probability of the drowsy and non-drowsy classes by fitting a logistic function to the engineered behavioural features. Although Logistic Regression does not explicitly model temporal dependencies, it provides an important benchmark against which the performance of more sophisticated temporal learning models can be assessed.

#### 3.6.2. Random Forest

Random Forest was adopted as a nonlinear ensemble learning algorithm capable of capturing complex interactions among the engineered behavioural features. The classifier constructs multiple decision trees using bootstrap aggregation and random feature selection, thereby improving classification robustness while reducing the risk of overfitting. Random Forest has been widely applied in intelligent transportation and driver behaviour analysis because of its ability to model nonlinear relationships without extensive parameter tuning. In this study, the classifier served as a strong machine learning baseline for comparison with the recurrent neural network models.

#### 3.6.3. Long Short-Term Memory Network

Long Short-Term Memory (LSTM) networks were implemented to learn temporal relationships within the behavioural eye sequences. Unlike conventional feedforward neural networks, LSTM incorporates memory cells and gating mechanisms that enable information to be retained over extended time intervals while mitigating the vanishing gradient problem associated with traditional recurrent neural networks. The network receives a sequence of engineered behavioural feature vectors and learns temporal patterns associated with the gradual transition from alertness to drowsiness. A dropout layer was incorporated after the LSTM layer to reduce overfitting, followed by a fully connected hidden layer employing the Rectified Linear Unit (ReLU) activation function. The final output layer utilised a sigmoid activation function to produce the probability of driver drowsiness.

#### 3.6.4. Bidirectional Long Short-Term Memory Network

Bidirectional Long Short-Term Memory (BiLSTM) extends the conventional LSTM architecture by processing temporal sequences in both forward and backward directions. This bi-directional learning mechanism enables the network to exploit preceding and succeeding observations within the already observed temporal window, without accessing frames beyond the final prediction time. Such an approach has the potential to improve the representation of behavioural fatigue patterns where eye movement dynamics may depend on relationships among observations occurring earlier and later within the temporal window. Similar to the LSTM implementation, dropout regularisation and fully connected dense layers were incorporated before the final sigmoid output layer. The performance of the BiLSTM model was subsequently compared with the classical machine learning models and the unidirectional LSTM under identical experimental conditions.

**Figure 5:**
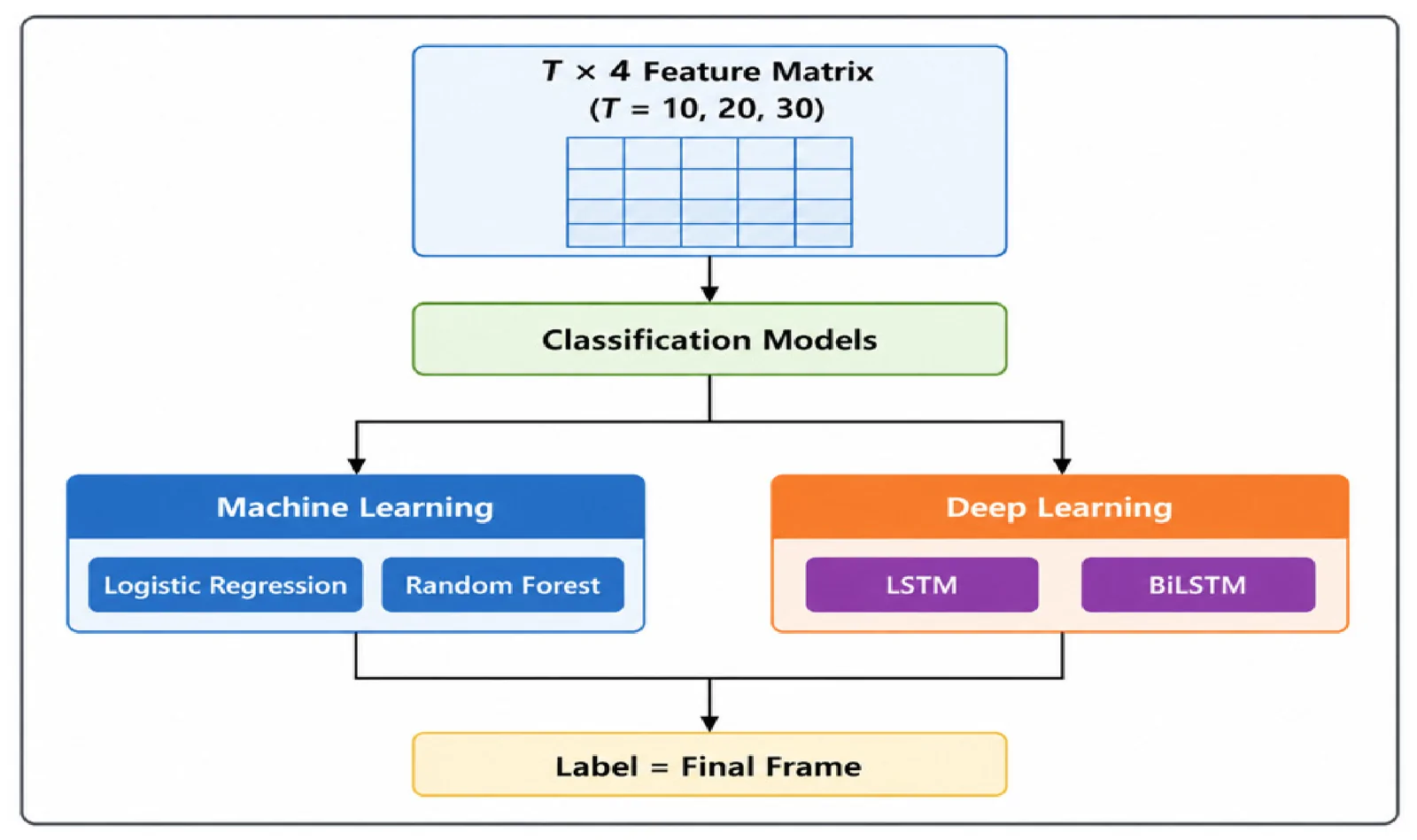
Investigated classification models.

The four classification models investigated in this study represent two complementary learning paradigms. Logistic Regression and Random Forest were selected as representative classical machine learning classifiers, whereas LSTM and BiLSTM were employed to investigate the benefits of temporal deep learning for behavioural fatigue detection. Figure 5 summarises the classification models evaluated and their categorisation within the proposed framework.

As illustrated in Figure 5, the experimental design facilitates a balanced comparison between conventional machine learning and recurrent deep learning approaches under identical feature representations and evaluation conditions. This comparative framework enables the influence of model complexity on subject-independent behavioural fatigue detection to be systematically investigated while ensuring that performance differences are attributable to the classification algorithms rather than variations in data preprocessing or validation strategy.

### 3.7. Model Training and Experimental Configuration

All experiments were implemented in Python (3.12.3, 2024) using the Scikit-learn and TensorFlow/Keras machine learning libraries. The classical machine learning models, namely Logistic Regression and Random Forest, were implemented using the Scikit-learn framework whereas the Long Short-Term Memory (LSTM) and Bidirectional Long Short-Term Memory (BiLSTM) networks were developed using TensorFlow with the Keras application programming interface (API). The experiments were conducted within the Anaconda scientific computing environment to facilitate library management and reproducibility.

To ensure consistency across all experiments, identical temporal sequences generated using the procedure described in Section 3.4 were supplied to each classification model. Three sequence lengths comprising 10, 20, and 30 consecutive frames were investigated to evaluate the influence of temporal context on behavioural fatigue recognition. For each sequence length, model training and testing were performed independently under the Leave-One-Driver-Out (LODO) validation protocol described in Section 3.5.

The LSTM and BiLSTM networks received as input a sequence of four engineered behavioural features consisting of the Eye Aspect Ratio (EAR), Rolling Mean EAR, EAR Delta, and Eye Closure Indicator. Each recurrent network contained 64 hidden memory units followed by a dropout layer with a dropout rate of 0.30 to minimise overfitting. The extracted temporal representation was subsequently passed through a fully connected dense layer comprising 32 neurons with Rectified Linear Unit (ReLU) activation. An additional dropout layer with a rate of 0.20 was applied before the final sigmoid output neuron, which estimated the probability of driver drowsiness.

Model optimisation was performed using the Adam optimiser with binary cross-entropy as the objective function. Training was conducted using a batch size of 64 samples, while early stopping based on validation loss was employed to prevent unnecessary training and reduce the likelihood of overfitting. Within each LODO fold, the data from the three development drivers were further partitioned into internal training and validation sets by assigning complete recording streams to the validation set, targeting approximately 15% of the development observations. This stream-disjoint partitioning ensured that overlapping temporal windows derived from the same recording stream could not occur in both the internal training and validation sets. The MinMaxScaler was fitted exclusively on the internal training partition and subsequently applied unchanged to the internal validation and held-out test partitions. The held-out driver therefore remained completely unseen during model development. Early stopping used a patience of seven epochs and restored the model weights corresponding to the lowest validation loss; consequently, the number of training epochs varied across folds according to model convergence.

Class imbalance was handled according to the training mechanism of each classifier. Logistic Regression and Random Forest used balanced class weighting during fitting, whereas the LSTM and BiLSTM models were trained with unweighted binary cross-entropy on the observed class distribution. This distinction was retained as part of the predefined model-specific configurations rather than introducing post hoc weighting to the recurrent models. Given the moderate overall class distribution (54.64% drowsy and 45.36% non-drowsy), evaluation emphasised class-sensitive metrics, including precision, recall, F1-score, AUROC, and AUPRC, in addition to accuracy.

For reproducibility, a fixed random seed of 42 was adopted for the primary experiments to initialise the Python, NumPy, and TensorFlow random number generators. In addition, the stability of the stochastic recurrent models was assessed for the selected 30-frame temporal configuration using three independent random seeds (42, 123, and 2026). The same LODO folds, stream-disjoint internal validation procedure, and training-only feature scaling were retained across these runs. Stability was assessed by first averaging performance across the four held-out drivers for each seed and then computing the mean and standard deviation of these LODO-averaged results across the three seeds. Table 4 summarises the principal experimental parameters adopted in this study.

Following the configuration presented in Table 4, all of the classification models were trained and evaluated using the same behavioural feature representation, temporal sequence lengths, and LODO partitions. Maintaining consistent data preparation and evaluation conditions ensured that observed performance differences reflected the characteristics of the respective classifiers rather than differences in dataset composition or validation strategy. This consistent experimental design therefore provides a fair basis for comparing conventional machine learning and temporal deep learning approaches for behavioural driver fatigue detection.

### 3.8. Performance Evaluation Metrics

The predictive performance of the classification models was evaluated using multiple complementary performance metrics in order to provide a comprehensive assessment of behavioural fatigue detection under subject-independent conditions. Since no single evaluation metric adequately characterises classifier performance, this study employed Accuracy, Precision, Recall, F1-score, Area under the Receiver Operating Characteristic Curve (AUROC), and Area under the Precision–Recall Curve (AUPRC). Collectively, these metrics assess overall classification performance, class-specific prediction capability, discrimination ability, and robustness across varying decision thresholds.

The classification performance was computed independently for each Leave-One-Driver-Out evaluation fold and subsequently averaged across all four test subjects to obtain an overall estimate of model generalisation. This evaluation strategy ensures that the reported performance reflects the ability of each classifier to recognise fatigue patterns in previously unseen drivers rather than memorising subject-specific behavioural characteristics. The selected evaluation metrics and their respective interpretations are summarised in Table 5.

As shown in Table 5, each evaluation metric provides complementary information regarding classifier performance. Accuracy offers an overall measure of predictive correctness but may not fully reflect the effectiveness of fatigue detection when class distributions are unequal. Precision evaluates the proportion of predicted drowsiness events that are correctly identified, thereby reflecting the reliability of system-generated fatigue warnings. Conversely, Recall measures the proportion of actual drowsiness events successfully detected by the classifier and is particularly important in intelligent driver monitoring systems because missed fatigue events may have serious road safety consequences.

The F1-score provides a balanced assessment by simultaneously considering Precision and Recall, making it suitable for comparing models when both false alarms and missed detections are undesirable. In addition to these threshold-dependent metrics, AUROC evaluates the ability of a classifier to discriminate between drowsy and non-drowsy states over all possible decision thresholds, whereas AUPRC provides additional insight into model performance when class frequencies are not perfectly balanced. Employing these complementary evaluation measures therefore enables a more comprehensive comparison of the classical machine learning and deep learning models investigated in this study.

The processed EAR dataset is provided in Appendix A; the image-level processing and data-preparation notebooks are provided in Appendix B; the machine-learning model codes are provided in Appendix C; the EAR-threshold sensitivity-analysis code is provided in Appendix D; and the controlled-baseline-analysis code is provided in Appendix E.

## 4. Experimental Results and Discussion

### 4.1. Experimental Overview

This section presents the experimental evaluation of the proposed behavioural driver drowsiness detection framework under a subject-independent validation protocol. The objective is to assess the effectiveness of the engineered behavioural features and temporal sequence modelling in recognising drowsiness patterns across previously unseen subjects.

Four classification models comprising two conventional machine learning algorithms (Logistic Regression and Random Forest) and two recurrent deep learning architectures (LSTM and BiLSTM) were evaluated using the Leave-One-Driver-Out (LODO) protocol described in Section 3.5. Experiments were conducted using three temporal sequence lengths (T = 10, T = 20, and T = 30) to investigate the influence of temporal context on detection performance.

Performance was assessed using Accuracy, Precision, Recall, F1-score, Area under the Receiver Operating Characteristic Curve (AUROC), and Area under the Precision–Recall Curve (AUPRC). Unless otherwise stated, reported results represent the average performance obtained across the four independent test subjects, thereby providing an unbiased estimate of subject-independent generalisation.

The results are organised to provide a comprehensive evaluation of the proposed framework. First, the performance of the four classification models is compared under the Leave-One-Driver-Out evaluation protocol. This is followed by analyses of temporal sequence length, repeated-run stability of the recurrent models, the contributions of temporal context and feature engineering, subject-wise performance, the selected configuration for drowsiness detection, EAR-threshold sensitivity, confusion matrix behaviour, and comparison with previous studies.

### 4.2. Comparative Performance of the Classification Models

The predictive performance of the four classification models was evaluated under the subject-independent Leave-One-Driver-Out (LODO) protocol using three temporal sequence lengths (10, 20, and 30 frames). Model performance was assessed using Accuracy, Precision, Recall, F1-score, Macro F1-score, Weighted F1-score, AUROC, and AUPRC. For each configuration, the reported results represent the mean and standard deviation obtained across the four independent test subjects. The comparative performance of all evaluated models is presented in Table 6.

#### Performance Comparison

Table 6 reveals that classifier performance varied across both model type and temporal sequence length. Overall, the classical machine learning models demonstrated more consistent performance than the recurrent deep learning models under the adopted subject-independent evaluation protocol. Among the evaluated configurations, Logistic Regression achieved the highest mean accuracy, with its best value of 0.576 obtained at a sequence length of 10 frames. However, Random Forest with a sequence length of 30 frames achieved the highest drowsy-class F1-score of 0.618 and the highest AUPRC of 0.663, together with a drowsy class recall of 0.701. These results show that the relative performance of the evaluated configurations depends on the evaluation metric: Logistic Regression provided the highest overall classification accuracy, whereas Random Forest at 30 frames achieved the highest drowsy-class F1-score and AUPRC among the evaluated configurations. This indicates that higher overall classification accuracy did not necessarily correspond to better performance on drowsy-class measures, highlighting the importance of considering complementary performance metrics beyond accuracy when assessing behavioural fatigue detection systems.

The recurrent neural network models demonstrated competitive recall values for the drowsy class but generally produced lower overall classification accuracy than the classical machine learning models. Their response to sequence length was metric-dependent, with longer windows improving some discrimination measures but not producing consistent gains in accuracy or drowsy-class F1-score. This pattern suggests that the four engineered behavioural features may provide limited temporal complexity for exploiting the full representational capacity of recurrent deep learning architectures.

Among the evaluated Random Forest configurations, performance improved with increasing sequence length, culminating in the highest observed drowsy class F1 score at T = 30. The favourable precision–recall balance achieved at this sequence length supports its selection for further analysis in Section 4.7.

### 4.3. Effect of Temporal Sequence Length on Behavioural Fatigue Detection

The selection of temporal sequence length plays an important role in determining the amount of behavioural information available for fatigue recognition. While shorter sequences capture immediate eye movement patterns, longer sequences provide a richer temporal context that may better represent the gradual progression of driver drowsiness. To investigate this effect, three sequence lengths comprising 10, 20, and 30 consecutive frames were evaluated across all four classification models under identical subject-independent experimental conditions.

The results presented in Table 6 indicate that the influence of temporal sequence length was model dependent. Longer observation windows improved several performance measures for Random Forest, whereas their effects were metric-dependent for BiLSTM and less consistent for Logistic Regression and LSTM. Random Forest showed its strongest drowsy class performance at a sequence length of 30 frames, achieving its highest F1 score and AUPRC at this temporal window. This suggests that the usefulness of additional temporal information depends on the classification model and the performance measure considered, rather than longer sequences producing a uniform improvement across all models.

For the recurrent neural network models, the effect of increasing sequence length was inconsistent across the evaluation metrics and did not produce a systematic improvement in drowsy-class F1-score. Performance also varied across the held-out subjects, indicating that extending the temporal window alone does not necessarily translate into improved subject-independent classification when the input representation is limited to four engineered behavioural features.

Interestingly, the results also suggest that relatively short temporal windows already capture a considerable proportion of the behavioural information required for drowsiness detection. From a practical deployment perspective, this finding is important because longer sequences increase computational cost and inference latency while providing only incremental improvements in predictive performance. Consequently, the choice of sequence length should consider both detection accuracy and computational efficiency, particularly for real-time intelligent transportation systems.

The experimental findings indicate that the influence of temporal context on behavioural fatigue detection is dependent on both the underlying classifier and the discriminative capacity of the engineered behavioural features. The results therefore reinforce the importance of jointly considering feature representation, sequence length, and model characteristics rather than assuming that increasing temporal context will uniformly improve classification performance.

### 4.4. Repeated-Run Stability of the Recurrent Models

Because LSTM and BiLSTM training involves stochastic model initialisation and optimisation, an additional repeated-run analysis was conducted to assess the sensitivity of their performance to random seed selection. The selected T = 30 configuration was evaluated using three independent random seeds (42, 123, and 2026) while retaining the same LODO folds, stream-disjoint internal validation procedure, and training-only feature scaling. For each seed, performance was first averaged across the four held-out drivers, after which the mean and standard deviation across the three runs were calculated. The results are presented in Table 7.

The repeated-run results showed limited seed-to-seed variation in overall Accuracy, AUROC and AUPRC for both recurrent models. Accuracy exhibited standard deviations of approximately 0.004 for both LSTM and BiLSTM, while the standard deviations of AUROC and AUPRC remained at or below 0.005. Greater variability was observed for drowsy-class Recall and F1-score, with Recall standard deviations of 0.035 for LSTM and 0.039 for BiLSTM. These results indicate that overall discrimination performance was relatively stable across the three evaluated seeds, although class-specific performance showed greater sensitivity to stochastic training variation.

### 4.5. Controlled Analysis of Temporal Context and Feature Engineering

To isolate the contributions of temporal context and engineered behavioural features, an additional controlled baseline experiment was conducted using Random Forest under the same Leave-One-Driver-Out evaluation protocol. Four input configurations were compared: single-frame raw EAR (B1), single-frame engineered features comprising EAR, Rolling Mean EAR, EAR Delta, and Eye Closure Indicator (B2), 30-frame sequences containing raw EAR only (B3), and 30-frame sequences containing the four engineered features (B4). This design permits the effect of temporal context to be examined independently of feature engineering by comparing B1 with B3, while B1–B2 and B3–B4 comparisons indicate the additional contribution of the engineered features at the single-frame and temporal levels, respectively. The results of these controlled baseline comparisons are presented in Table 8.

The controlled comparison demonstrates that temporal context contributed more strongly to performance than feature engineering alone. When the input was restricted to raw EAR, extending the representation from a single frame (B1) to a 30-frame temporal sequence (B3) increased the mean accuracy from 0.514 to 0.553, drowsy-class F1-score from 0.531 to 0.618, AUROC from 0.532 to 0.624, and AUPRC from 0.547 to 0.663. This consistent improvement across the four measures provides direct evidence that incorporating preceding eye-behaviour observations adds discriminative information beyond instantaneous EAR measurements under unseen-driver evaluation.

Feature engineering also improved the single-frame representation, with B2 outperforming B1 in accuracy, drowsy-class F1-score, AUROC, and AUPRC. However, its incremental contribution became substantially smaller when temporal context was already available. The T = 30 engineered configuration (B4) produced only marginal differences relative to T = 30 raw EAR (B3): accuracy increased from 0.553 to 0.556 and AUROC from 0.624 to 0.626, while drowsy-class F1-score remained approximately 0.618 and AUPRC was essentially unchanged. Thus, the results indicate that the principal gain arose from incorporating temporal context, whereas the engineered features provided clearer additional value in the absence of a multi-frame temporal representation.

These findings complement the sequence-length results in Table 6 by distinguishing the effect of temporal aggregation from that of feature construction. They also show that the performance of the selected T = 30 Random Forest configuration cannot be attributed solely to the derived EAR features, since a comparable temporal raw-EAR baseline achieved similar performance. Accordingly, the contribution of the proposed representation is interpreted conservatively as a compact means of representing complementary aspects of eye behaviour rather than as an independently dominant source of predictive improvement.

### 4.6. Subject-Wise Performance Analysis

Unlike conventional random train–test partitioning, the Leave-One-Driver-Out (LODO) validation protocol provides a realistic assessment of a classifier’s ability to generalise to previously unseen subjects. Consequently, evaluating model performance across individual test subjects offers valuable insight into the robustness of the proposed behavioural fatigue detection framework under practical deployment conditions.

To examine inter-subject variability directly, Table 9 presents the fold-specific results for the selected Random Forest configuration at *T* = 30 and the predefined EAR threshold of 0.21. Each LODO fold corresponds to one driver being held out exclusively for testing while the remaining drivers are used for model development. The reported results therefore represent performance on each previously unseen driver rather than performance obtained from a random partition of sequences.

The subject-wise results revealed appreciable inter-driver variability despite the relatively narrow range of accuracy values. Drowsy-class recall ranged from 0.557 for Driver 002 to 0.943 for Driver 006, while AUROC ranged from 0.540 to 0.783. Driver 006 exhibited particularly high sensitivity to the drowsy class but lower precision (0.488), indicating a greater tendency to classify observations as drowsy. In contrast, Driver 002 produced the lowest recall and AUROC. These differences are consistent with the variability observed across the individual LODO folds and indicate that model behaviour varies meaningfully across unseen drivers (Table 9).

The matched always-drowsy baseline further contextualises the fold-specific accuracy results. Random Forest exceeded the corresponding baseline for Drivers 001 and 006 by 0.051 and 0.141, respectively, but remained below the baseline for Drivers 002 and 005 by 0.044 and 0.043. Across the four LODO folds, the mean always-drowsy baseline accuracy was 0.530, compared with the mean Random Forest accuracy of 0.556, representing an average improvement of approximately 0.026. The variation across drivers indicates that the improvement over a trivial class-frequency baseline was not consistent across unseen subjects and reinforces the need to interpret accuracy together with the class-sensitive and threshold-independent metrics.

The observed variability emphasises the importance of subject-independent evaluation when assessing behavioural fatigue detection systems. Importantly, although overlapping temporal windows increase the number of sequence samples available for model estimation, they do not increase the number of independent participants represented in the study. The present evaluation therefore remains based on four independent drivers, and the sequence count should not be interpreted as a substitute for greater participant diversity.

The LODO evaluation nevertheless enables the available subjects to be assessed under strict cross-subject separation, thereby avoiding subject overlap between training and testing. The observed inter-subject differences further suggest that future behavioural fatigue detection systems should be evaluated on larger and more diverse driver populations and may benefit from adaptive approaches capable of accommodating individual differences in fatigue-related eye behaviour.

### 4.7. Analysis of the Selected Configuration for Drowsiness Detection

Based on the emphasis of this study on identifying the drowsy class, Random Forest with a sequence length of 30 frames was selected for further analysis. Although Logistic Regression achieved the highest mean accuracy, the Random Forest configuration attained the highest drowsy class F1 score (0.6181) and AUPRC (0.6626), together with a drowsy class recall of 0.7008. These metrics are particularly relevant to the present application because they reflect the model’s ability to identify the safety-critical drowsy class while accounting for the balance between precision and recall. Table 10 summarises the performance of the selected configuration.

As shown in Table 10, the selected Random Forest configuration achieved a mean drowsy-class recall of 0.701 and F1-score of 0.618, although its mean accuracy remained modest at 0.556. Logistic Regression achieved higher overall classification accuracy, whereas Random Forest produced the highest drowsy-class F1-score and AUPRC among the evaluated configurations. The selection of the T = 30 Random Forest configuration for further analysis therefore reflects the study’s emphasis on examining drowsy-class detection behaviour rather than a claim of superior overall classification performance.

The results further indicate that Random Forest can capture nonlinear relationships within the compact EAR-based behavioural representation without requiring a recurrent architecture. However, its absolute performance under subject-independent evaluation remains limited, indicating that the four-driver dataset and the available behavioural features do not support strong claims regarding practical detection capability. The selected configuration is therefore examined primarily to characterise its behaviour under strict unseen-driver evaluation and to identify the limitations that remain for broader deployment.

### 4.8. Sensitivity to the EAR Eye-Closure Threshold

To assess whether the performance of the selected RF configuration depended materially on the fixed EAR threshold used to construct the Eye Closure Indicator, a sensitivity analysis was conducted at thresholds of 0.19, 0.20, 0.21, 0.22, and 0.23 while retaining the *T* = 30 sequence length and the same LODO evaluation protocol. Performance remained highly stable across the tested range. Mean accuracy varied only from 0.556 to 0.557, F1-score for the drowsy class from 0.617 to 0.619, AUROC from 0.625 to 0.627, and AUPRC from 0.662 to 0.663. These results in Table 11 indicate that the reported RF performance is not materially dependent on the specific 0.21 threshold within the evaluated range.

### 4.9. Confusion Matrix Analysis

**Figure 6:**
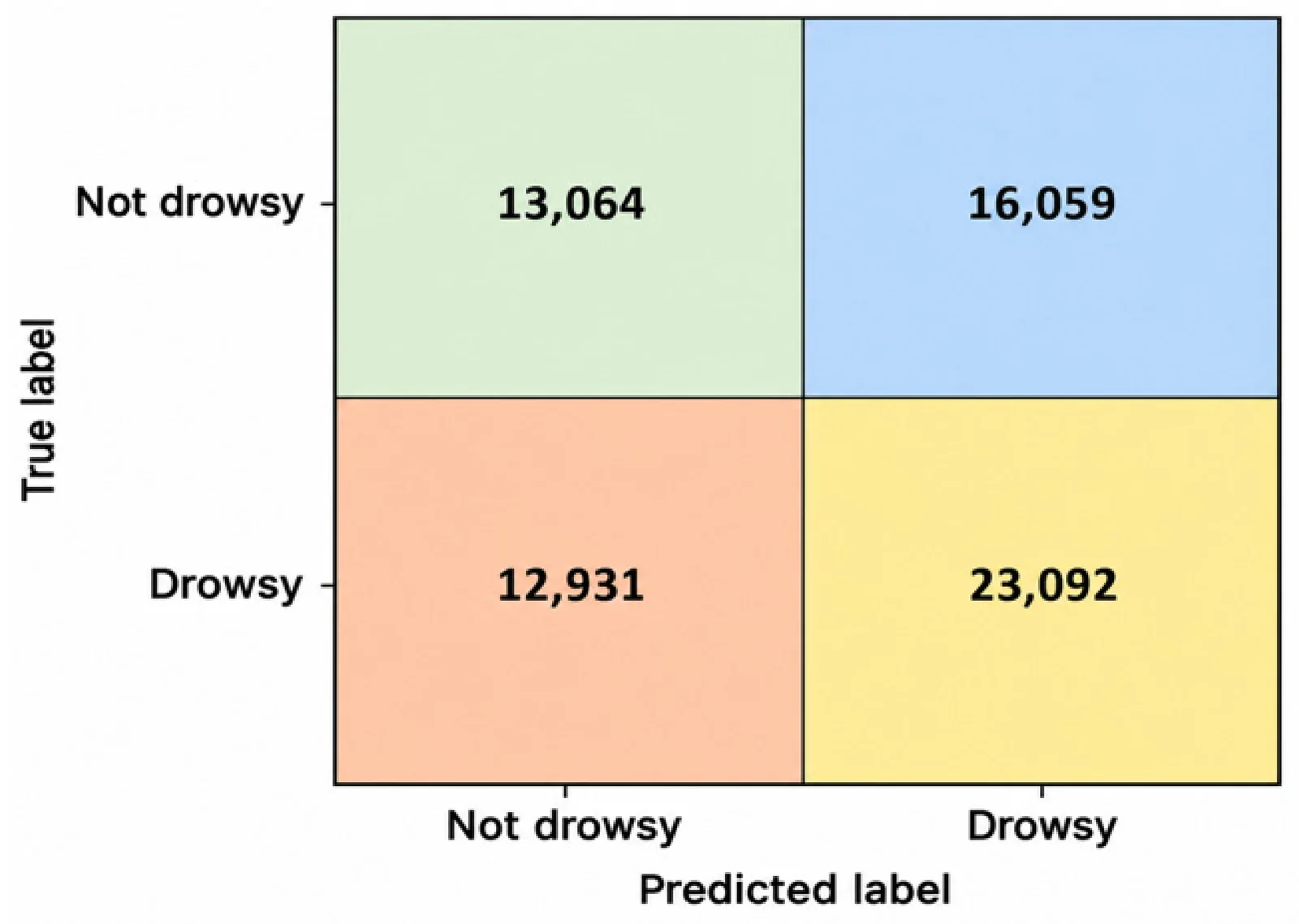
LODO confusion matrix for Random Forest @ *T* = 30.

To further examine the predictive behaviour of the selected Random Forest configuration, the confusion matrix obtained from the Random Forest classifier with a sequence length of 30 frames was analysed. Unlike aggregate evaluation metrics, the confusion matrix provides a detailed representation of correctly and incorrectly classified samples, thereby offering additional insight into the strengths and limitations of the proposed behavioural fatigue detection framework. Figure 6 presents the confusion matrix corresponding to the selected Random Forest configuration.

Because the confusion matrix aggregates predictions across all LODO test folds, metrics computed directly from the pooled counts may differ slightly from the mean fold level performance values reported in Table 10.

As illustrated in Figure 6, the classifier correctly identified 23,092 drowsy samples and 13,064 non-drowsy samples, while 12,931 drowsy samples were classified as non-drowsy and 16,059 non-drowsy samples were classified as drowsy. The pooled confusion matrix therefore yielded an accuracy of approximately 55.5% compared with the fold-averaged accuracy of 55.6% reported in Table 10. The small difference arises because Figure 6 pools predictions from all four held-out drivers before computing accuracy, whereas Table 10 reports the mean of the four fold-level accuracies. For context, an always-drowsy classifier applied to the same pooled test samples would achieve an accuracy of approximately 55.3%, corresponding to the proportion of drowsy samples in the pooled test set. Thus, the selected Random Forest configuration provides only a small improvement in pooled accuracy over this trivial baseline, illustrating why accuracy alone is insufficient for interpreting the present results.

The confusion matrix further shows that the classifier favoured sensitivity to the drowsy class at the expense of a substantial false-positive rate. Based on the pooled counts, 16,059 of the 29,123 non-drowsy samples were incorrectly classified as drowsy, corresponding to a false-positive rate of approximately 55.1%. At the same time, 23,092 of the 36,023 drowsy samples were correctly identified. The comparatively higher drowsy-class recall therefore reflects a detection trade-off rather than uniformly strong classification performance. This finding reinforces the need to interpret the F1-score and AUPRC alongside the false-positive behaviour and the modest overall accuracy when assessing the practical significance of the selected configuration.

Nevertheless, the presence of both false positive and false negative predictions indicates that behavioural eye features alone may not fully characterise all manifestations of driver fatigue under diverse operating conditions. Future studies incorporating complementary physiological, vehicular, or contextual information may further reduce classification errors while improving the robustness of subject-independent fatigue detection systems.

### 4.10. Discussion and Comparison with Previous Studies

The experimental findings demonstrate that model performance in behavioural drowsiness detection depends not only on classifier complexity, but also on the feature representation and evaluation protocol. Under the Leave-One-Driver-Out setting adopted in this study, Logistic Regression achieved the highest mean classification accuracy, whereas Random Forest with a sequence length of 30 frames achieved the highest drowsy-class F1-score and AUPRC among the evaluated configurations. This finding indicates that the nonlinear relationships contained in the derived EAR-based features could be captured effectively by an ensemble classifier without requiring a recurrent deep learning architecture.

The subject-independent design is central to the interpretation of these results. Drowsiness observations extracted from the same recording are temporally related while behavioural patterns such as baseline eye geometry, blinking frequency, and eyelid movement may also differ systematically between drivers. If overlapping sequences or neighbouring frames from one driver are randomly distributed across the training and test sets, the resulting evaluation may partly measure the recognition of participant-specific and recording-specific patterns rather than generalisation to an unfamiliar driver. Hasan et al. similarly observed that random and conventional fold-based partitions may allow highly correlated observations to occur in both the training and test sets. In their multimodal physiological study, Leave-One-Participant-Out validation was regarded as the most useful subject-independent procedure with Random Forest attaining an accuracy of 80.1%. The authors emphasised that the protocol produced more credible generalisation estimates because the test participant was not represented during training.

The greater difficulty of subject-independent evaluation is also evident in the results reported by [45]. Their hybrid EEG and eye-tracking model achieved 99.93% accuracy in intra-session evaluation, but its performance decreased to 88.67% across sessions and 78.19% across subjects. Comparable reductions occurred for the EEG-only, eye-tracking, and alternative multimodal models. This pattern illustrates why the performance values obtained in the present study should not be compared directly with very high accuracies reported under the image-level, frame-level or participant-dependent splits. The present Leave-One-Driver-Out results represent evaluation on entirely unseen individuals and therefore provide a more conservative but deployment-relevant assessment.

The metric-specific performance of Random Forest also shows that deep temporal models are not invariably advantageous when the input consists of a compact set of engineered behavioural descriptors. The four features used in this study encode instantaneous eye openness, short-term smoothing, temporal change, and threshold-based eye closure. Random Forest can model nonlinear interactions and threshold relationships among these variables while remaining comparatively insensitive to feature scaling and limited sample diversity. Recurrent models, in contrast, must estimate a larger number of parameters and may require greater driver diversity to learn temporal representations that transfer reliably across subjects. The experimental dataset contained only four available drivers; therefore, the LSTM and BiLSTM models may have learned temporal regularities that were insufficiently invariant to individual behavioural differences.

The sequence-length analysis nevertheless confirms that temporal context remains important. The best Random Forest result occurred with 30-frame sequences, suggesting that the longer observation window captured more stable information about sustained eye closure and evolving eyelid behaviour than the shorter 10- and 20-frame alternatives. However, the effect of increasing sequence length was not uniform across all classifiers. A longer temporal window provides more contextual evidence, but it may also increase redundancy and expose recurrent networks to driver-specific temporal patterns. Sequence length should therefore be treated as a model-dependent hyperparameter rather than assuming that longer windows will consistently improve performance.

The findings also have implications for the interpretation of existing behavioural drowsiness studies. High image-level accuracy can demonstrate the effective recognition of eye states or facial expressions, but it does not necessarily establish that the complete system will generalise to unfamiliar drivers. Dataset resources such as FatigueView [48] and manD 1.0 [49] increasingly emphasise participant diversity, multiple acquisition conditions, and multimodal driver states, reflecting the need to evaluate systems beyond narrowly controlled or participant-overlapping samples. Future evaluations of the present framework should therefore include a larger and more heterogeneous driver population, different camera conditions, and independent external datasets.

The contribution of the present study is not a claim that Random Forest universally outperforms LSTM or BiLSTM. Rather, the results show that under a compact engineered behavioural representation and a strict unseen driver protocol, Random Forest provided the strongest drowsy class F1 score and AUPRC among the evaluated configurations, while Logistic Regression achieved the highest mean accuracy. This finding underscores the importance of matching model complexity to dataset size, feature structure, and evaluation objective. It further highlights the importance of considering evaluation design when comparing drowsiness detection performance across studies.

## 5. Conclusions, Limitations and Future Research

This study investigated behavioural driver drowsiness detection under strict subject-independent evaluation, with particular attention to the influence of temporal context and classifier choice on generalisation to previously unseen drivers. Four engineered eye-based features comprising Eye Aspect Ratio, Rolling Mean EAR, EAR Delta, and Eye Closure Indicator were organised into temporal sequences of 10, 20, and 30 frames and evaluated using Logistic Regression, Random Forest, LSTM, and BiLSTM classifiers. Unlike conventional frame level evaluation, the Leave-One-Driver-Out protocol ensured complete separation between the training and test drivers, thereby providing a more realistic assessment of model performance under unseen driver conditions.

The experimental findings indicate that under the evaluated conditions, increased model complexity does not necessarily translate into stronger generalisation when behavioural information is represented by a compact set of engineered eye features. Logistic Regression achieved the highest mean classification accuracy, while Random Forest at 30 frames achieved the highest drowsy-class F1 score and AUPRC among the evaluated configurations. The recurrent LSTM and BiLSTM models did not consistently outperform the classical classifiers despite their ability to model sequential dependencies. Temporal sequence length nevertheless influenced model behaviour, with the 30 frame configuration providing the most favourable operating point for Random Forest. These findings reinforce the importance of considering feature representation, temporal context, and evaluation protocol alongside model architecture when developing driver monitoring systems.

The study is limited by the availability of only four drivers (001, 002, 005, and 006) in the downloaded NTHU-DDD source subset used for the experiments and by its reliance exclusively on behavioural eye-based features. Subjects 003 and 004 were not present in the downloaded source files used in this study and were therefore not excluded on the basis of preprocessing outcomes or model performance. Although the adopted LODO protocol strengthens the credibility of the reported generalisation performance, the limited number of subjects restricts the extent to which the findings can be generalised to broader driver populations and operating environments. The modest absolute classification performance observed under strict LODO evaluation further indicates that the four behavioural eye features alone may not provide sufficient discriminative information for robust generalisation across heterogeneous unseen drivers. In addition, eye behaviour alone may not capture all manifestations of fatigue, particularly where individual differences, illumination, facial occlusion or other driving conditions affect behavioural observations.

Although the sensitivity analysis indicated stable performance across EAR thresholds of 0.19–0.23, the Eye Closure Indicator remains dependent on a fixed threshold and may not fully accommodate subject-specific variations in eye characteristics and acquisition conditions. In addition, the framework has not yet been externally validated on an independent driver dataset.

Future research should therefore extend the framework to larger and more diverse driver populations and evaluate its performance across independent datasets and real driving environments. Incorporating complementary behavioural, physiological, vehicular, or contextual information may further improve robustness while retaining the subject independent evaluation principle adopted in this study. Further investigation of adaptive and personalised decision thresholds may also help accommodate natural differences in eye behaviour among drivers.

Overall, the findings suggest that under the conditions examined in this study, realistic evaluation and careful behavioural representation are fundamental to the development of reliable driver drowsiness detection systems, and that comparatively simple classifiers can remain competitive when assessed under rigorous unseen driver conditions.

## Figures and Tables

**Table 1:** Summary of the behavioural dataset used for model development.

Characteristic	Value
Dataset	NTHU Driver Drowsiness Detection (NTHU-DDD)
Subjects in Experimental Dataset	4 (001, 002, 005, 006)
Total Image Frames	65,929
Video Streams	27
Drowsy Frames	36,023 (54.64%)
Non-drowsy Frames	29,906 (45.36%)
Validation Strategy	Leave-One-Driver-Out (LODO)

**Table 2:** Distribution of samples across the four available subjects.

Subject	Non-Drowsy	Drowsy	Total
001	9336	9578	18,914
002	7829	10,595	18,424
005	8843	13,087	21,930
006	3898	2763	6661
**Total**	**29,906**	**36,023**	**65,929**

**Table 3:** Behavioural features extracted from each image frame.

Feature	Description	Purpose
Eye Aspect Ratio (EAR)	Geometric measurement of eye openness computed from facial landmarks	Measures instantaneous eye state
Rolling Mean EAR	Moving average of EAR over five consecutive frames	Reduces noise and captures gradual temporal changes
EAR Delta	Difference between consecutive EAR values	Captures blink dynamics and rapid eye movement changes
Eye Closure Indicator	Binary feature generated using an EAR threshold of 0.21	Represents eye closure state associated with fatigue

**Table 4:** Experimental configuration adopted for model training.

Parameter	Value
Programming Language	Python
Development Environment	Anaconda
Classical ML Library	Scikit-learn
Deep Learning Library	TensorFlow/Keras
Input Features	EAR, Rolling Mean EAR, EAR Delta, Eye Closure Indicator
Sequence Lengths	10, 20, 30
LSTM/BiLSTM Hidden Units	64
Dense Layer	32 neurons (ReLU)
Dropout Rates	0.30 and 0.20
Output Activation	Sigmoid
Optimiser	Adam
Loss Function	Binary Cross-Entropy
Maximum Epochs	50
Batch Size	64
Early Stopping	Validation loss, patience = 7, restore best weights
Internal Validation	Stream-disjoint validation, approximately 15% of LODO development data
Feature Scaling	MinMaxScaler fitted on internal training partition only
Logistic Regression	max_iter = 1000, class_weight = balanced
Random Forest	300 trees, class_weight = balanced
Validation Strategy	Leave-One-Driver-Out (LODO)
Primary Random Seed	42
Stability Analysis Seed	42, 123, 2026

**Table 5:** Performance evaluation metrics adopted in this study.

Metric	Mathematical Expression	Purpose
Accuracy	(TP + TN)/(TP + TN + FP + FN)	Measures the proportion of correctly classified samples.
Precision	TP/(TP + FP)	Measures the reliability of predicted drowsiness events.
Recall	TP/(TP + FN)	Measures the ability to correctly identify drowsy drivers.
F1-score	2(PR)/(P + R)	Balances Precision and Recall.
AUROC	Area under ROC curve	Measures overall discrimination capability across thresholds.
AUPRC	Area under Precision–Recall curve	Evaluates classifier performance under moderate class imbalance.

**Table 6:** Comparative performance of the evaluated classification models under subject-independent LODO validation.

*Sequence Length*	*Model*	*Accuracy*	*Precision (Drowsy)*	*Recall (Drowsy)*	*F1 (Drowsy)*	*Macro F1*	*Weighted F1*	*AUROC*	*AUPRC*
*10*	BiLSTM	0.535 ± 0.030	0.566 ± 0.078	0.647 ± 0.241	0.580 ± 0.054	0.521 ± 0.033	0.521 ± 0.027	0.609 ± 0.141	0.626 ± 0.038
*10*	LSTM	0.535 ± 0.036	0.563 ± 0.089	0.671 ± 0.219	0.592 ± 0.044	0.520 ± 0.043	0.519 ± 0.050	0.628 ± 0.132	0.630 ± 0.040
*10*	Logistic Regression	0.576 ± 0.072	0.608 ± 0.061	0.592 ± 0.235	0.579 ± 0.096	0.570 ± 0.075	0.571 ± 0.069	0.633 ± 0.122	0.629 ± 0.036
*10*	Random Forest	0.537 ± 0.017	0.558 ± 0.061	0.674 ± 0.177	0.596 ± 0.030	0.525 ± 0.010	0.527 ± 0.008	0.595 ± 0.102	0.623 ± 0.052
*20*	BiLSTM	0.527 ± 0.035	0.567 ± 0.067	0.608 ± 0.272	0.556 ± 0.077	0.514 ± 0.030	0.511 ± 0.022	0.626 ± 0.128	0.611 ± 0.024
*20*	LSTM	0.569 ± 0.086	0.614 ± 0.054	0.571 ± 0.283	0.560 ± 0.126	0.557 ± 0.091	0.559 ± 0.084	0.628 ± 0.129	0.623 ± 0.027
*20*	Logistic Regression	0.574 ± 0.075	0.609 ± 0.065	0.589 ± 0.241	0.577 ± 0.100	0.567 ± 0.078	0.569 ± 0.071	0.641 ± 0.130	0.641 ± 0.044
*20*	Random Forest	0.549 ± 0.012	0.571 ± 0.066	0.688 ± 0.175	0.609 ± 0.026	0.537 ± 0.007	0.539 ± 0.012	0.614 ± 0.106	0.646 ± 0.059
*30*	BiLSTM	0.504 ± 0.028	0.556 ± 0.091	0.623 ± 0.263	0.554 ± 0.050	0.482 ± 0.062	0.475 ± 0.079	0.645 ± 0.139	0.652 ± 0.056
*30*	LSTM	0.502 ± 0.039	0.557 ± 0.096	0.624 ± 0.261	0.554 ± 0.044	0.475 ± 0.084	0.467 ± 0.103	0.643 ± 0.136	0.646 ± 0.050
*30*	Logistic Regression	0.574 ± 0.076	0.612 ± 0.071	0.591 ± 0.247	0.578 ± 0.102	0.567 ± 0.078	0.568 ± 0.072	0.647 ± 0.135	0.651 ± 0.050
*30*	Random Forest	0.556 ± 0.012	0.577 ± 0.069	0.701 ± 0.173	0.618 ± 0.025	0.542 ± 0.010	0.544 ± 0.019	0.626 ± 0.107	0.663 ± 0.062

**Table 7:** Repeated-run stability of the recurrent models at T = 30 across three random seeds.

*Model*	*Accuracy*	*Precision (Drowsy)*	*Recall (Drowsy)*	*F1-Score (Drowsy)*	*AUROC*	*AUPRC*
*LSTM*	0.505 ± 0.004	0.557 ± 0.003	0.662 ± 0.035	0.569 ± 0.017	0.646 ± 0.004	0.652 ± 0.005
*BiLSTM*	0.505 ± 0.004	0.556 ± 0.001	0.641 ± 0.039	0.560 ± 0.023	0.646 ± 0.002	0.651 ± 0.003

**Table 8:** Controlled Random Forest baseline comparison under subject-independent LODO evaluation.

*Configuration*	*Accuracy*	*F1-Score (Drowsy)*	*AUROC*	*AUPRC*
*B1: Single-frame Raw EAR*	0.514 ± 0.012	0.531 ± 0.013	0.532 ± 0.030	0.547 ± 0.061
*B2: Single-frame Engineered*	0.529 ± 0.017	0.573 ± 0.019	0.561 ± 0.059	0.576 ± 0.039
*B3: T = 30 Raw EAR*	0.553 ± 0.012	0.618 ± 0.021	0.624 ± 0.108	0.663 ± 0.064
*B4: T = 30 Engineered*	0.556 ± 0.012	0.618 ± 0.025	0.626 ± 0.107	0.663 ± 0.062

**Table 9:** Subject-wise performance of Random Forest at *T* = 30 and matched always-drowsy baseline under LODO evaluation.

*Held-Out Driver*	*Test Sequences*	*Accuracy*	*Always-Drowsy Accuracy*	*Δ Accuracy*	*Precision (Drowsy)*	*Recall (Drowsy)*	*F1 (Drowsy)*	*AUROC*	*AUPRC*
*001*	18,682	0.564	0.513	+0.051	0.559	0.704	0.623	0.589	0.592
*002*	18,192	0.538	0.582	−0.044	0.614	0.557	0.584	0.540	0.632
*005*	21,698	0.560	0.603	−0.043	0.645	0.599	0.622	0.594	0.696
*006*	6574	0.561	0.420	+0.141	0.488	0.943	0.644	0.783	0.731

**Table 10:** Performance of the selected configuration under subject-independent LODO evaluation.

Performance Measure	Value
Classification Model	Random Forest
Sequence Length	30
Mean Accuracy	0.556 ± 0.012
Mean Precision (Drowsy)	0.577 ± 0.069
Mean Recall (Drowsy)	0.701 ± 0.173
Mean F1-score (Drowsy)	0.618 ± 0.025
Mean AUROC	0.626 ± 0.107
Mean AUPRC	0.663± 0.062

**Table 11:** Sensitivity analysis results.

*EAR Threshold*	*Accuracy*	*F1-Score (Drowsy)*	*AUROC*	*AUPRC*
*0.19*	0.557 ± 0.013	0.619 ± 0.026	0.626 ± 0.109	0.663 ± 0.063
*0.20*	0.556 ± 0.013	0.617 ± 0.027	0.627 ± 0.107	0.663 ± 0.063
*0.21*	0.556 ± 0.012	0.618 ± 0.025	0.626 ± 0.107	0.663 ± 0.062
*0.22*	0.556 ± 0.011	0.618 ± 0.025	0.625 ± 0.106	0.662 ± 0.062
*0.23*	0.557 ± 0.012	0.618 ± 0.026	0.625 ± 0.106	0.662 ± 0.062

## Data Availability

The data used in this study were obtained from the NTHU Driver Drowsiness Detection (NTHU-DDD) dataset. Supporting materials and analysis code generated for this study are accessible through the repository links provided in the appendices.

## Appendix A. Main Dataset (EAR)

https://github.com/ajayioo/tut_fsati_fatigue_data/blob/main/eye_features.csv (accessed on: 23 September 2026).

## Appendix B. Image-Level Processing and Data Preparation

https://github.com/ajayioo/tut_fsati_fatigue_data/blob/main/ear_image_process_function.ipynb (accessed on: 23 September 2026).

https://github.com/ajayioo/tut_fsati_fatigue_data/blob/main/perclos_batch_process_image.ipynb (accessed on: 23 September 2026).

## Appendix C. Jupyter Python ML Model Codes for the Study

https://github.com/ajayioo/tut_fsati_fatigue_data/blob/main/Fatigue_Behavioral_Clean_Rerun.ipynb (accessed on: 23 September 2026).

https://github.com/ajayioo/tut_fsati_fatigue_data/blob/main/Fatigue_Behavioral_Clean_Rerun_for_Reviewer2_Comment.ipynb (accessed on: 23 September 2026).

## Appendix D. Jupyter Python Sensitivity Analysis Codes

https://github.com/ajayioo/tut_fsati_fatigue_data/blob/main/EAR_threshold_sensitivity_RF_T30_LODO.ipynb (accessed on: 23 September 2026).

## Appendix E. Jupyter Python Controlled Baseline Analysis Codes

https://github.com/ajayioo/tut_fsati_fatigue_data/blob/main/rf_controlled_baseline.ipynb (accessed on: 23 September 2026).
